# Factors affecting the provision of high-quality postnatal care services in Zanzibar: a qualitative study

**DOI:** 10.1186/s12884-023-06035-0

**Published:** 2023-10-06

**Authors:** Anna Öjendal, Herborg Holter, Helen Elden, Sanura Salim, Malin Bogren

**Affiliations:** 1 Sahlgrenska Academy, University of Gothenburg, Gothenburg, Sweden; 2 Institute of Health and Care Sciences, Sahlgrenska Academy, University of Gothenburg, Gothenburg, Sweden; 3 Mnazi Mmoja Hospital, Zanzibar, Tanzania

**Keywords:** Postnatal care, Maternal health, Maternal mortality, Low-income countries, Sub-Saharan Africa

## Abstract

**Background:**

In Sub-Saharan Africa, the postnatal period is associated with high mortality and accounts for a substantial proportion of maternal deaths. Although postnatal care has been identified as critical in reducing maternal mortality, the quality of care provided is often inadequate. Tanzania and Zanzibar have not made sufficient progress towards achieving the Sustainable Development Goals on maternal health, and there is limited knowledge about the utilization and quality of postnatal follow-up. The aim of this study was therefore to explore factors affecting the provision of high-quality postnatal care services in the urban area of Zanzibar.

**Methods:**

Five focus group discussions were performed in Swahili with 25 healthcare providers from primary healthcare units in urban Zanzibar. Interviews were audio-recorded, transcribed verbatim, translated into English, and analysed using qualitative content analysis with an inductive approach.

**Results:**

Factors affecting provision of high-quality postpartum care services could be divided into three generic categories. *Difficulty achieving high attendance* comprised three subcategories: long waiting times, low awareness among women, and out-of-pocket payment. *Lack of basic resources* also comprised three subcategories: shortage of healthcare providers, lack of adequate space, and inadequate medical equipment. *Insufficient care routines* comprised two subcategories: lack of guidelines and deficient chain of information.

**Conclusions:**

The present findings suggest that the women’s perceptions of postnatal care do not align with the intended purpose of routine postnatal care. Instead, the postnatal period primarily leads to visits to health facilities only when urgent care is required, and there is a lack of awareness about the importance of postnatal care. Moreover, limited resources, including equipment, staff, and space, as well as long waiting times, hinder the delivery of high-quality care and contribute to a negative reputation of postnatal care services. To effectively reach all women and improve postnatal care, it is necessary to increase basic resources, modify health education approaches, and enhance the flow of information between different levels of care using context-specific strategies.

**Supplementary Information:**

The online version contains supplementary material available at 10.1186/s12884-023-06035-0.

## Background

Maternal and newborn health outcomes continue to be a major concern worldwide, particularly in low- and middle-income countries where maternal mortality rates and preventable newborn deaths remain high. Despite the critical role that postnatal care plays in improving these outcomes, the quality of care provided is often inadequate. An integrative systematic review of postnatal care in Africa from 2021 found that none of the examined study sites incorporated all the standards in the World Health Organization (WHO) guidelines for postnatal care [1]. The WHO has recognized the importance of improving postnatal care, and recently published updated recommendations to enhance essential and routine postnatal care for women and newborns [2]. These recommendations include maternal and newborn care as well as health systems and health promotion interventions. However, the problem of inadequate postnatal care attendance and low-quality care persists globally, particularly in Sub-Saharan Africa [3, 4]. Addressing these challenges is crucial in improving maternal and newborn health outcomes and achieving the Sustainable Development Goals for good health and well-being [5].

Maternal mortality rates in Sub-Saharan Africa are high, with a level of 542 women per 100,000 observed in 2017 [6]. Approximately 60% of maternal deaths in Sub-Saharan Africa occurred during the postnatal period in 2013 [7]. Additionally, neonatal deaths account for 47% of under-5 mortality globally, with sub-optimal breastfeeding contributing to more than a third of postneonatal deaths. This further highlights the importance of high-quality postnatal care services, including support and counselling for breastfeeding [8]. Several factors contribute to low postnatal care attendance rates in Sub-Saharan Africa, including low education levels, young age, rural residence, home births, lack of occupation, low exposure to media, and lack of antenatal care visits [3, 9].

The importance of respectful care, including woman-centred care, informed choice, and kindness from healthcare providers, has been highlighted in the literature as an influencing factor in low- and middle-income countries. For example, a recent review of postnatal care in Sub-Saharan Africa emphasized the significance of such factors [10]. Furthermore, a study in Malawi showed that negative experiences with healthcare providers during antenatal care can lead to low postnatal attendance [11]. Although recent studies have focused on postnatal care from the perspective of patients, less attention has been given to the perspective of healthcare providers [10, 12].

The maternal health situation in Tanzania and Zanzibar is similar to other sub-Saharan countries, with a maternal mortality rate of 424 deaths per 100,000 live births observed in 2017 [13]. Approximately 44.3% of maternal deaths in Tanzania between 2005–2010 occurred during the postnatal period [14]. Barriers to postnatal care attendance in rural Tanzania include poor awareness of the importance of care, poor availability of care, ambivalence over the quality of care, and cultural traditions [15–17]. The 2022 Tanzania Demographic Health Survey reported that the rate of postnatal attendance within 48 h was 59% in Zanzibar [18]. Moreover, the 2021 Zanzibar Health Bulletin reported that postnatal attendance within 48 h among health facility births was 84% in Zanzibar and 61% in Mjini (the western urban area), and that 12% of women who gave birth at a health facility completed four postnatal visits [19]. The most common problems identified during the postnatal period were severe postpartum haemorrhage, sepsis, and anaemia [19].

Maternal and reproductive health is a pressing issue in low- and middle-income countries, and the postnatal period is a key factor in the well-being of the mother and child [1, 7, 8, 14, 20]. There are several studies on life-threatening obstetric emergencies in Zanzibar [21–23] as well as a recent study on postpartum family planning [24], but there are limited data on the utilization and quality of postnatal follow-up at the primary healthcare clinics in Zanzibar. The aim of this study was to explore factors affecting the provision of high-quality postnatal care services in the urban area of Zanzibar. Hence, the findings are expected to contribute to areas of improvement and useful knowledge regarding the implementation of postnatal care in Zanzibar and in similar settings in Tanzania and Sub-Saharan Africa.

## Methods

### Study design and settings

A qualitative study was conducted, utilizing focus group discussions (FGDs) with healthcare providers working in primary healthcare units connected to the referral hospital Mnazi Mmoja in Zanzibar. This method is deemed suitable when attempting to gain insight into a relatively unexplored topic [25], such as factors affecting the provision of high-quality postpartum care services in Zanzibar.

Maternal healthcare is free of charge in Zanzibar. Secondary or tertiary levels of care are responsible for providing childbirth and comprehensive post-abortion care, although some of the primary healthcare units also provide childbirth services [26]. The units vary in size, capacity, and distance from the referral hospital Mnazi Mmoja, and offer postnatal care from 48 h to 42 days after childbirth. Three of them also provide childbirth services. In 2021 there were roughly 52 000 live births at all health facilities in Zanzibar, including primary, secondary, and tertiary levels of care. This is estimated to account for approximately 70% of all births in Zanzibar [19]. The primary healthcare units were chosen in dialogue with the district office in consideration to the number of employees involved in postnatal care.

### Participants and data collection

Focus Group Discussions (FGDs) were conducted during the third quarter of 2022. The study received written approval from the district office and oral permission from the managers of the primary healthcare units to conduct the FGDs. The native-speaking author (SS) approached the operational manager at each unit to inform them about the study and seek permission to recruit healthcare providers for FGDs related to postnatal care. Eligible healthcare providers were identified and invited to participate if they met the inclusion criteria: being a midwife, medical doctor, or assistant nurse, being at least 18 years of age, and being employed at one of the primary healthcare units. These healthcare providers were selected as they were considered to have the most experience and insight regarding the phenomenon: factors affecting the provision of high-quality postnatal care services.

A total of 25 individuals agreed to participate in the study: 23 midwives, one medical doctor, and one assistant nurse. All participants were Muslim and female. Their ages ranged from 20 to 69 years (mean: 42 years) and their working experience ranged from less than 1 year to 39 years (mean: 15 years).

Before the start of each FGD, the participants received written and oral information in Kiswahili about the study, its purpose, and the voluntary nature of their participation. They were asked to avoid disclosing the information shared within the group, in order to protect the confidentiality of the other members. Five FGDs were conducted with 3–6 healthcare providers in each group at the participants’ workplaces. The timing of the FGDs was decided in communication with the healthcare providers to minimize disturbance of the daily work. The FGDs were performed in Kiswahili by one of the authors (SS) in a private area with closed doors and were based on an interview guide containing open questions to answer the overall research question: what factors affect the provision of high-quality postnatal care services. The interview guide is presented in Additional File 1. The interview guide was tested in the initial FGD, and since no adjustments were deemed necessary, data from this interview was included in the analysis Interviews were audio-recorded and lasted between 22 and 39 min (mean: 31 min).

### Data analysis

The interviews were transcribed verbatim and translated into English. Inductive content analysis was performed as inspired by Elo and Kyngäs [25]. First the text was read carefully and thoroughly to gain familiarity with the content. Next, the data were organized by open coding, and meaning units corresponding to the aim of the study were assembled and coded. The coded meaning units were then compared and grouped into subcategories and generic categories. This analysis was conducted by AÖ, in close collaboration with MB and SS, until full agreement was reached. The final phase of the analysis involved all authors. An example of the data analysis process is presented in Table 1.

**Table 1:** Example of the process of the inductive content analysis

**Meaning unit**	**Code**	**Subcategory**	**Generic category**
*“Therefore, it becomes a problem because there’s less privacy and she doesn’t feel comfortable.” (FGD 4)*	Lack of privacy	Lack of adequate space	Lack of basic resources
*“When the women come here, she should check heamoglobin but the machine isn’t there.” (FGD 1)*	Insufficient tests	Inadequate medical equipment	Lack of basic resources

## Results

Three generic categories were found to cover the factors affecting the provision of postnatal care in the urban area of Zanzibar: *difficulty achieving high attendance, lack of basic resources*, and *insufficient care routines*. These categories and their eight subcategories are given in Table 2. Selected quotations from the FGDs are presented in the results to exemplify each subcategory [25], each labelled with the number of the relevant FGD.

**Table 2:** Generic categories and subcategories describing factors affecting provision of postnatal care

**Generic category**	**Subcategory**
Difficulty achieving high attendance	Long waiting times
	Low awareness among women
	Out-of-pocket payment
Lack of basic resources	Shortage of healthcare providers
	Lack of adequate space
	Inadequate medical equipment
Insufficient care routines	Lack of guidelines
	Deficient chain of information

### Difficulty achieving high attendance

#### Long waiting times

Healthcare providers from all FGDs pointed out long waiting times as a reason for women not to attend routine postnatal care services. They described their impressions of women feeling bored, tired, or as if they were wasting their time. Staff shortages, lack of equipment, lack of examination space, and low prioritization of postnatal care were underlying reasons for long waiting times.

The problem is right there because every time she comes, she sees a queue which breaks her heart. Sometimes she comes eagerly, but when she sees a big queue, she turns back and goes away. (FGD 3)

Additionally, fragmented care packages leading to multiple queues for vaccination, postnatal care, and contraceptives were described.

When she comes here, she finds another queue; sometimes you can tell that people who come to get vaccination for their children also want to get services, but they see another big queue, they’re disappointed, and they leave. (FGD 3)

Several strategies were proposed to manage the issue of long waiting times, including prioritising postnatal patients, offering scheduled appointments, or having assigned dates for postnatal care. The idea was also expressed of developing a complete postnatal care package, meaning an earmarked supply of tests and equipment including all services that the mother and newborn should be provided with according to recommendations, to avoid multiple visits and queues.

#### Low awareness among women

Awareness regarding postnatal care services was another challenge in achieving increased attendance. There was an overall perception that most mothers used postnatal care services only when an urgent visit was needed, and not as a routine procedure. However, removal of stitches was described as a routine procedure, and most of these women would attend at least one visit. Other reasons for postnatal visits were pain or suspected infection in the genitals.

They come to close the wound, but if someone gives birth and doesn’t have that problem then she doesn’t come; she says “Why should I go when I’ve given birth safely?” (FGD 4)

Some healthcare providers felt that if they emphasized the need for postnatal care services then the mothers did listen and came back, while others had found that the mothers were not receptive to health education. Women who chose to give birth at home were also a group in need of further health education, but were hard to reach.

There was a day here we got a case of a mother, the mother gave birth at home, so she didn’t go to the hospital, she had a tear but when we asked her to look at her, she refused. All the advice we gave, she didn’t want to listen. (FGD 5*)*

There were some misconceptions that the postnatal care services were only important for the newborn, which sometimes led to the mother deciding to stay at home.

And here maybe there’s a difficulty of understanding, because when a mother comes here, in their understanding the goal is the initial vaccinations for the child, and so you’ll find that the child is brought by some other relative from the household. (FGD 4)

The healthcare providers also gave several examples of misconceptions regarding what would be included in the postnatal exams and how they would be performed. They had experienced some mothers being reluctant to attend postnatal care because of examination of their private parts.

Now when they come here, they think that everyone who comes gets a speculum, we provide more education, but when you go, your whole body is examined and there’s the possibility of being examined in your private parts, because a woman is examined all over, and especially women who had stitches must be examined, but if she didn’t get stitches then it’s not important to look at her private parts, but their minds are on inserting a speculum. (FGD 1)

They had also observed a few cases where the mother’s apprehension of postnatal care services included the idea of compulsory contraceptives. Overall, the combination of lacking knowledge and misperceptions created a situation where the mothers who were severely ill were more likely to attend postnatal care services.

Several ideas were suggested to improve awareness and attendance of postnatal care, including health education delivered via information programs on the TV and radio, outreach services with community health visitors who could motivate mothers to attend, and sending appointment reminders via text messages. Some of the healthcare providers also thought that husbands could play an important role by encouraging the mothers to attend postnatal care. The importance of word of mouth was emphasized repeatedly, and a good reputation was perceived as key in improving attendance.

#### Out-of-pocket payment

The necessity of payment emerged as another reason for women not to receive care. The healthcare providers’ perception of problems related to out-of-pocket payment included both women who could not afford to pay for the services and women who were reluctant to pay for postnatal care services due to disappointment at not being offered the services free of charge, as they had expected.

You’re telling her that you want to refer her to Mnazi Mmoja, and you have to tell her to donate 10 thousand for ambulance fuel because the ambulance here doesn’t have fuel. Sometimes it’s a problem, then they tell you, ahh, we don’t have the money, for us it’s a challenge. (FGD 2)

Yes, some people say that they can’t afford it, so they get used to having free services. If you mention money or ask them to pay for the test then they aren’t ready to come, they say: “ahh I have already given birth safely, what can I do?” (FGD 1)

The possible need for payment not only contributed to low attendance but also to low quality of care, since the healthcare providers were not able to offer or implement the postnatal care services as recommended.

### Lack of basic resources

#### Shortage of healthcare providers

There was a large variation between the different primary healthcare units in terms of both number of personnel and the personnel categories. The consequences of staff shortage differed depending on the size of the primary healthcare units and their offered services. For some of the facilities, staff shortage was the main explanation for long waiting times, while some considered the main consequence of staff shortage to be obstructing the possibility of providing good-quality care. Examples of parts of the postnatal care routine which were deprioritized because of time shortage were breastfeeding counselling, testing of haemoglobin level, and taking blood pressure.

On the side of staff, there’s so few that this is the first big challenge, because you have to divide each section with one staff member, [...], so you wanted to serve pregnant women and then go to others, [...] you need one person to serve them, so that you don’t keep them waiting for a long time, but it’s impossible. (FGD 5)

#### Lack of adequate space

In some settings where the staffing was adequate, a lack of space instead emerged as the primary hindrance to providing care. Some of the healthcare providers explained that the poor condition of the facility was a challenge to both attendance and ability to provide good care. One of them said:

But that area is small, so even if there’s two of you [midwives], you can only handle one client. (FGD 3)

Several healthcare providers described how the lack of a private space, or even the inability for the women to cover themselves, made the women uncomfortable during physical examinations.

She doesn’t have a rubber sleeping mat or even a kanga^1^, maybe to cover her, when you do the assessment. So there’s a problem because there’s less privacy and she doesn’t feel comfortable. (FGD 4)

The lack of privacy caused problems in giving health education related to sexual and reproductive health.

Sometimes you want to educate them, but there are a lot of men around; you can’t even say other things because you don’t want them to hear. (FGD 2)

#### Inadequate medical equipment

A lack of medical equipment often meant that the healthcare providers had no possibility of providing adequate care. Examples included haemoglobin testing machines, urine strip tests, blood pressure machines, thermometers, gloves, and sutures. Broken machines, lack of batteries, and broken beds were also mentioned.

Even the medical equipment is challenging, like the examination bed, it’s so bad and she [the woman] is so tired that you’re afraid to even lift her up, you think she’ll fall; so the fear of risking injury is also a challenge. (FGD 5)

Because of the equipment shortage, the only alternative was to advise the mothers to go to a private clinic. However, this led to both lower patient attendance and increased waiting times.

If there’s no equipment, we’ll tell her to check other private hospitals, because often those services are available, so you tell her to check this test and that and then bring the results here. Some come back and bring the results, and others don’t go for the check up and just go their own way. (FGD 2)

The healthcare providers also discussed difficulties regarding oversight of the facilities and the system of supply request, which were responsibilities of the district office. A coordinator was meant to inspect the health facilities in order to detect challenges, but these inspections were often delayed and so the challenges were not addressed in time. The system of supply request meant that they were only able to order a certain amount of equipment, regardless of the number of patients, and each order was followed by a period when they could not put in any additional orders.

But it isn’t time yet to request more, because when you’re given equipment, you must wait for a certain period, you aren’t allowed to ask for something else, so it ends up like that. Also, like the pregnant mother’s package, there’s no such thing, they’re only brought together. (FGD 5)

### Insufficient care routines

#### Lack of guidelines

It became clear that the guidelines for postnatal care were not used, and in some cases were not actually known about, which subsequently affected the quality of the care. The standard procedure for postnatal care was described as being done solely out of habit. This meant that the quality of care relied on the experience and competence of individual healthcare providers, and inexperienced midwives had less resources to provide good-quality care. Moreover, updates to the recommendations on postnatal care were not distributed in a uniform way and were therefore not implemented in a uniform way either, regardless of the experience of the healthcare providers. Only one facility reported that guidelines existed in their health facility, but they had encountered other difficulties such as the material being written in English or being too long to easily use while providing care.

Let’s not lie, there’s no quality of postnatal care here and there are no guidelines that are followed. (FGD 1)

#### Deficient chain of information

The healthcare providers described deficiencies in the chain of information between different levels of care. Two aspects were discussed when transferring patients between care levels. First, the record book^2^ used as a medical record was insufficient, leading to a lack of information about the patient’s medical history. Second, referring patients from primary care to tertiary care could be challenging, since information about how and where the patient should be referred was lost along the way.

The biggest challenge we face first is that we don’t have the record book; the mother is here for you to handle, but there’s no record book, so you might have the wrong data. (FGD 1)

## Discussion

In this study from Zanzibar, three generic categories were found to cover the factors affecting the provision of high-quality postnatal care. The first category, *difficulty achieving high attendance*, described reasons why women chose not to seek postnatal care services. The second category, *lack of basic resources*, included lack of staff, equipment, and space that affected the quality of care, and had additional consequences in the form of decreased attendance due to unsatisfactory services. Finally, the organizational challenge of *insufficient care routines* made it hard to achieve improvement of care, for example due to a lack of guideline distribution.

To understand and discuss the findings, we use the three-delay model developed by Thaddeus and Maine [27]. The first delay is *delay in decision to seek care*, which is affected by financial situation, previous experience, severity or character of illness, and opinions of family*.* Factors identified with the first delay in this study were relevant to our first category, difficulty in achieving high attendance. The results showed low awareness of postnatal care among mothers as a challenge to attendance. According to our informants, women perceived postnatal care services as a service for urgent care and not as a routine procedure, which supports previous findings in rural areas in Tanzania [16, 17].

Inadequate knowledge of women’s reproductive health was also highlighted as a challenge that delayed care-seeking in a recent study from urban Zanzibar on postpartum contraceptives [24]. Maternal health information-seeking behaviour has been studied in rural Tanzania, revealing that most of the health information is obtained from non-professional health workers and family members. Furthermore, using written information was seen to be difficult, due to low education and husbands’ unwillingness to support their wives on maternal health issues [28]. This also supports the concept of a good reputation among peers as central to increasing attendance, as was observed both in this study and in a recent study on postpartum contraceptives in urban Zanzibar [24]. In the light of this, there is a call for broader and context-specific public health education to improve awareness.

Our study found that some mothers chose to stay home while someone else took their newborn to the health facility for postnatal care. This delay in seeking timely postnatal care is consistent with previous research suggesting that postnatal care is often viewed as only necessary for the newborn, and that the mother’s health is neglected [15]. By staying home, the mother may miss vital follow-up care during a vulnerable time period, given the high maternal mortality within the first two days after childbirth [29]. This results in a discontinuity of care for the mother.

The second delay is *delay in reaching the health facility* [27] due to distance, inadequate roads, the cost of transportation, and the distribution of health facilities. This was not reported as a challenge in the urban setting of our study, which is in contrast to previous studies on postnatal care conducted in rural areas in Tanzania [15–17] and other Sub-Saharan countries such as Uganda, Nigeria, and Zambia [30–32] where transport has been observed as an essential barrier to maternal health services. Reasons for the differing results of this study may include shorter distances and the availability of public transport during the daytime.

The third delay, *delay in receiving adequate and appropriate care and treatment at the health facility* [27], is due to a lack of necessities including supply of equipment and medicine, available well-trained staff, and a well-functioning referral system. In the present study, scarcity of resources such as testing supply, medicine, and examination beds made it impossible to provide postnatal care in accordance with the midwives’ training. For example, staff shortage was widely reported, and this factor has previously been stated to have associations with several unwanted outcomes such as higher incidence of postpartum haemorrhage and decrease in exclusive breastfeeding [33, 34]. Work stress among nurses has also been suggested to inhibit compassion in giving care and adequate nurse-patient communication [35], possibly leading to poor experience of care and unsatisfying health education. It is obvious from the findings of this study that the organizational challenges of care are intertwined, and the insufficiencies affect each other. The finding of lack of basic resources also played a part in the difficulty of achieving a higher attendance at postnatal care visits. The attempts to improve care were unsatisfactory; for example, inspections of the health facilities and distribution of guidelines were delayed or did not occur.

Another interesting finding is the lengthy waiting times for mothers to receive care, as this is an important factor which affects attendance, but which has not been emphasized in previous studies. This delay could be part of the first delay for mothers who do not attend, and the third delay for those who do attend. In low- and middle-income countries, patients are often targeted by multiple care programmes which can cause fragmentation of care and dissatisfaction as well as being time consuming for the patients [36]. This was found to be one of the reasons for not attending postnatal care services in the present study.

This study shows that postnatal care faces the challenge of providing care for two persons during the same period, which according to our informants affected both attendance and waiting times. The findings suggest the use of a postnatal care package that includes care for both the mother and newborn, integrated with other care packages such as immunization, nutrition, and HIV programmes. This could facilitate a continuum of care and be more time-efficient for the healthcare providers. Context-specific postnatal care packages, such as outreach services and outpatient care, have been suggested to improve attendance rates [36]. Home births are associated with low postnatal care attendance [3], but outreach services can help these mothers. A digital community health volunteer programme was implemented in Zanzibar from 2016–2019, including 10 out of 11 regions. A larger proportion of facility-based births and an increase in attending one postnatal care visit within 7 days from 34.8% to over 90% was shown among the enrolled women [37]. This suggests that outreach services paired with well-organized care packages could improve the continuum of care and increase attendance rates by targeting waiting times, the experience of care, and low awareness among mothers.

**Fig. 1:**
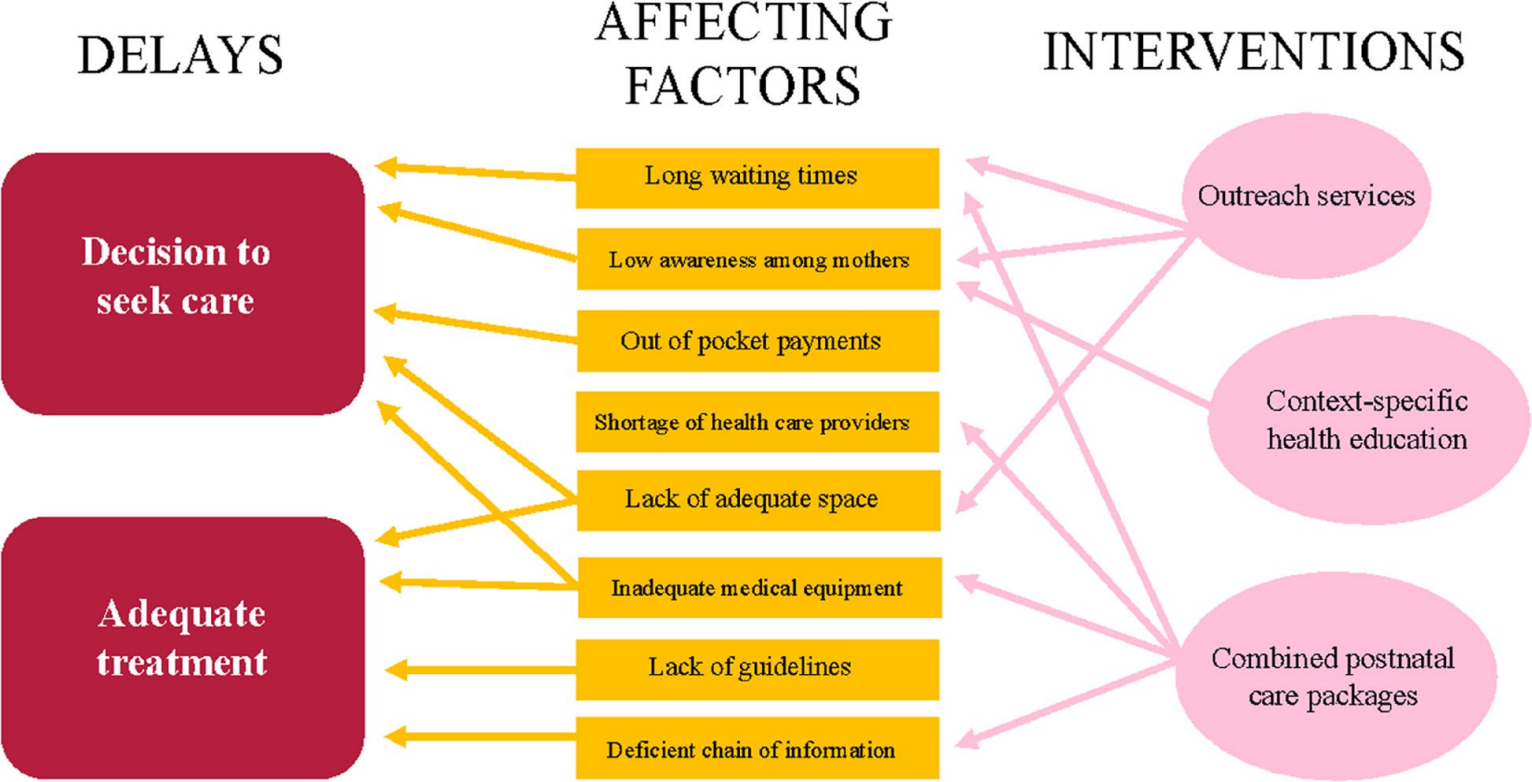
An illustration of how an intervention model could look inspired by the three-delay model. Note that this is only a visualization with examples from the findings of this study

The result of this study can contribute to the knowledge of postnatal care implementation in low- and middle-income settings and provide possible strategies to improve postnatal care in similar settings. However, in this local setting the findings could be of more direct value, as the factors found to affect the provision of high-quality postnatal care services could be the basis for an intervention study using the three-delay framework. Figure 1 illustrates an example of how an adapted model for this could look.

## Methodological considerations

The concepts of credibility, dependability, and transferability were taken into consideration to increase the trustworthiness of the study [38]. FGDs were conducted, which allowed for discussion and exploration of different perspectives. A well-established analytical method was used to increase the credibility of the study. The interviews were conducted by a native-speaking midwife who was familiar with the proceedings and the social and cultural norms, which facilitated the trust of the participants. The native speaking midwife was not employed at a healthcare unit and was not in a management position of the participants. The study explores factors affecting provision of postnatal care as experienced by healthcare providers, which should be considered strengthen the credibility. The experience of postnatal care from the perspective of the women are not explored in the study. The conditions of the topic were slightly different from their ordinary state due to renovations of health facilities, which could have affected the dependability of the data over time. The study did not include extensive quantitative data on postnatal attendance, complications, and treatments in the area, which could have provided a more detailed account of the challenges. Finally, the study focused on a specific setting and context, which limits the transferability of the findings to other settings; however, background information on the healthcare system and the characteristics of the participants have been included to facilitate comparisons.

## Conclusions

The findings of this study reveal interlinking factors that contribute to difficulty in providing high-quality postnatal care in the urban area of Zanzibar. The perceptions of postnatal care among women are not aligned with the intended purpose of routine postnatal care visits as a health-preservation function. Instead, women in the postnatal period visit a health facility only when in need of urgent care, and knowledge of the importance of postnatal care is not widespread. Lack of equipment, staff, and space obstructs high-quality care and gives postnatal care services a bad reputation among the mothers. To ensure comprehensive coverage of postpartum care for all women, it is important to enhance basic resources, provide context-specific health education, and improve information dissemination between different levels of care using context-specific strategies. This study also shows that the updated and newly-published WHO recommendations for maternal and newborn care [2] are not being followed. Future research studies are suggested to further explore the spectrum of postnatal complications in the area, in order to achieve a clearer picture of the needs of the women.

### Supplementary Information

**Additional file 1.**

## Data Availability

The dataset used in this study is available from the corresponding author on reasonable request.

## Abbreviations

FGD: Focus group discussion
WHO: World Health Organization

## Footnotes

1 Rubber sleeping mats were sometimes used as personal protective equipment instead of a disposable paper cover for the examination bed. A kanga is a piece of thin cotton clothing, similar to a sarong.

2 Each patient had their own handwritten notebook which they brought with them and which was used as a medical record.
