# Supplementary material for: Factors affecting the provision of high-quality postnatal care services in Zanzibar: a qualitative study
Source: BMC Pregnancy Childbirth. 2023 Oct 6;23:714. doi: 10.1186/s12884-023-06035-0 (PMC10559537; doi:10.1186/s12884-023-06035-0)
Supplement: Supplementary file 1 — Additional file 1. [file 12884_2023_6035_MOESM1_ESM.docx]

**Additional file 1**

## Title of data: Interview guide, English version

**Question 1:** Please tell me about your work here at the healthcare facility.

**Possible follow-up questions:**

1. What is included in a routine postnatal visit?

**Question 2:** What is the easiest part of providing postpartum care?

**Possible follow-up questions:**

1. Can you give an example?

2. Do you have another example?

**Question 3:** What is the most challenging part of providing postpartum care?

**Possible follow-up questions:**

1. Can you give an example?

2. Do you have another example?

**Question 4:** How important do the mothers seem to think postpartum visits are?

**Possible follow-up questions:**

1. If not very important, why do you think that is the case?

**Question 5:** What do you think are the reasons for not attending postpartum visits?

**Possible follow-up questions:**

1. Can you give an example?

2. Do you have another example?

**Question 6:** What do you need to provide high-quality postpartum care?

**Possible follow-up questions:**

1. Can you give an example?

2. Do you have another example?

**Question 7**: Do you think the postnatal care services provided in your area are enough? If not, why?

**Question 8:** Is there anything else you would like to add?
